# Coherent ultra-violet to near-infrared generation in silica ridge waveguides

**DOI:** 10.1038/ncomms13922

**Published:** 2017-01-09

**Authors:** Dong Yoon Oh, Ki Youl Yang, Connor Fredrick, Gabriel Ycas, Scott A. Diddams, Kerry J. Vahala

**Affiliations:** 1 T. J. Watson Laboratory of Applied Physics, California Institute of Technology, Pasadena, California 91125, USA; 2 Time and Frequency Division, National Institute of Standards and Technology, Boulder, Colorado 80305, USA

## Abstract

Short duration, intense pulses of light can experience dramatic spectral broadening when propagating through lengths of optical fibre. This continuum generation process is caused by a combination of nonlinear optical effects including the formation of dispersive waves. Optical analogues of Cherenkov radiation, these waves allow a pulse to radiate power into a distant spectral region. In this work, efficient and coherent dispersive wave generation of visible to ultraviolet light is demonstrated in silica waveguides on a silicon chip. Unlike fibre broadeners, the arrays provide a wide range of emission wavelength choices on a single, compact chip. This new capability is used to simplify offset frequency measurements of a mode-locked frequency comb. The arrays can also enable mode-locked lasers to attain unprecedented tunable spectral reach for spectroscopy, bioimaging, tomography and metrology.

## Abstract (web summary)

Continuum generation in optical fibres has enabled many applications, like optical frequency combs. Here, Oh *et al*. demonstrate controlled dispersive-wave generation in on-chip silica waveguides, which could have a similar impact on integrated devices.

Continuum generation in microstructured optical fibre^1^ gained importance with the generation of broad spectra^2^ for application in optical frequency combs. There, a mode-locked laser is broadened as a precurser to *f*-2*f* measurement of the comb offset frequency^3^ and self-referencing^4^. The advent of frequency microcombs^5^ and most recently the demonstration of femtosecond pulse generation in microcavities^6^,^7^,^8^,^9^ has focused attention on techniques for supercontinuum generation on a chip. Beginning with studies of Raman and four-wave-mixing effects in monolithic waveguides^10^, there has been steady progress towards supercontinuum generation using a variety of on-chip waveguide materials^11^,^12^,^13^,^14^,^15^,^16^,^17^. Low pulse energies for efficient broadening have been demonstrated on account of large nonlinear coefficients combined with nano-scale waveguide cross sections^14^,^15^, and devices operating in the mid-infrared^18^,^19^ are possible. Moreover, self-referencing has been achieved using a silicon nitride based, monolithic waveguide on silicon^20^.

However, while there has been remarkable progress on visible^21^,^22^ and ultraviolet band^23^,^24^,^25^ continuum generation in microstructured optical fibres, as well as harmonic ultraviolet and deep ultraviolet generation in gas-filled fibres^26^,^27^, there have been no reports of chip-based ultraviolet generation. Furthermore, widely tunable emission in a single device is a feature of monolithic arrays that has no parallel in optical fibres. Arrays enable precisely targeted wavelength emission for optimal self-referencing. As shown here, their compactness also eliminates the need for delay lines in these systems. Finally, this class of devices can be combined with microcombs and compact mode-locked lasers to provide user-designed coherent, short pulse light for optical clocks^28^, laser cooling^29^, quantum manipulation of atoms and ions^30^, and bioimaging^31^.

After describing the silicon ridge waveguide array fabrication process, measurements of dispersive-wave generation are presented that include a study of the phase matching wavelength dependence upon the waveguide geometry. Application of the waveguide array to optimized self-referencing of a mode-locked Yb fibre laser is then presented. Here, the dispersive wave phase matching wavelength is precisely matched to the 2*f* frequency so as to provide maximum signal-to-noise in the frequency-comb offset-frequency measurement. Also, the approach eliminates the delay line used in the standard self-referencing method. Finally, the continuum spectra produced by the waveguide arrays are analysed and modelled in detail.

## Results

### Silica ridge waveguide arrays

Continuum generation in waveguides results from a combination of nonlinear processes^10^,^32^. Self-phase modulation in combination with second-order anomalous dispersion induces temporal compression of an input pulse. As the pulse spectrally broadens, higher-order dispersion and Raman interactions become important and the pulse undergoes soliton fission. The resulting series of fundamental solitons experiences Raman self-frequency shifting towards longer wavelengths. If the soliton spectrum has significant overlap with spectral regions that feature normal dispersion, then it can radiate energy into a dispersive wave that phase matches to the soliton phase^33^. The dispersive wave, which can also be understood in terms of an analogy to Cherenkov radiation^34^, provides a powerful way to both engineer the spectral extent of the resulting optical continuum and to also spectrally concentrate optical power in new bands^22^,^35^. The waveguides demonstrated here apply lithographic control to engineer the generation of coherent ultraviolet to visible dispersive-wave radiation.

**Figure 1:**
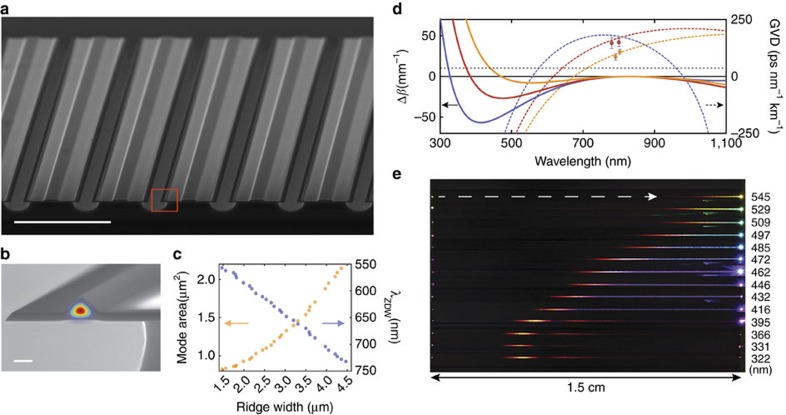
Phase matching condition and direct observation of dispersive wave generation in silica ridge waveguides. (**a**,**b**) Scanning electron microscope images of an array of silica ridge waveguides on a silicon chip. Silicon pillars support silica layers containing waveguides. The red box in **a** contains a silica waveguide whose cross section is shown in **b**; the cross section shows the calculated mode profile of the TM mode at wavelength 830 nm superimposed. Scale bar, 100 μm for (**a**) and 1 μm for (**b**). (**c**) Calculated mode area and zero dispersion wavelength (*λ*_ZDW_) are plotted versus the ridge base width. (**d**) Calculated group velocity dispersion (GVD, dashed lines) and phase-matching parameter Δ*β* (solid lines) for dispersive wave generation in TM polarization are plotted versus wavelength. Blue, red and yellow solid and dashed lines correspond to mode areas of 0.83, 1.03, 1.69 μm^2^ respectively. The phase matching condition Δ*β*=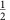
*γP*_p_ (830 nm pump) is satisfied at the intersections of the coloured solid lines and the black dashed line. The points with error bars are measured dispersion values obtained from sets of ten scans of spectral fringes measured using a Mach-Zehnder interferometer (see Methods). (**e**) Ultraviolet–visible dispersive wave generation in a silicon chip containing an array of waveguides with varying mode area (322 nm emission has area of 0.83 μm^2^ and 545 nm emission has area of 2.09 μm^2^). Image of multiple photographs of scattered light taken from above the 1.5 cm long chip in which infrared pulses are launched at the left side of each waveguide. Pulse energies are set to the threshold pulse energy for dispersive wave generation. The colour emission at the left side of the image is dispersive wave emission that has been reflected at the far right facet of the chip. The initial spectral broadening of the input pulse can be seen as the orange-red emission that gradually shifts diagonally upward right. The visible scattered light shown in the figure is a very small amount of the total generated. The vast majority of the light is forward propagating and collected into a multimode fibre (not shown).

**Figure 2:**
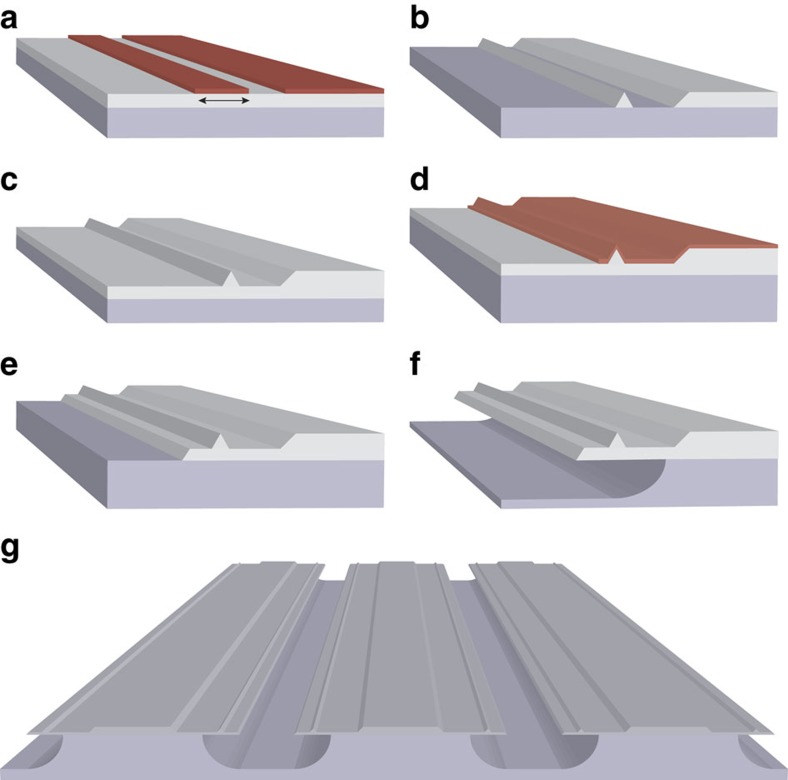
Ridge waveguide array microfabrication process. (**a**) Photolithography on thermal silica layer. Mask width is denoted by the double-arrow line. (**b**) HF wet-etching to define silica ridge. (**c**) Additional oxide layer is grown by thermal oxidation. (**d**) Supporting structure is patterned by photolithography. (**e**) HF wet-etching creates striped openings in the silica layer. (**f**) Isotropic etching of the silicon (XeF_2_) is performed to undercut the silica layer. (**g**) Rendering of final ridge waveguide array structure. Dependence of ridge dimension on mask width is shown in Supplementary Fig. 1.

The waveguide geometry is shown in Fig. 1a,b and features a silica ridge design that is air-clad on three sides. The air-cladding enables a high level of optical confinement to both increase the optical nonlinearity and shift the wavelength for zero group velocity dispersion towards visible wavelengths. The fabrication of the waveguides is an adaptation of the process used for dispersion engineering in high-*Q* resonators^36^. The process flow is presented in Fig. 2. More detail on the fabrication process is provided in Supplementary Note 1. The waveguides support both transverse electric (TE) and transverse magnetic (TM) polarizations. The TM-polarization provides dispersive wave generation at shorter wavelengths.

Waveguide arrays of varying mode area (0.76–2.22 μm^2^) were fabricated on the silicon chip. The effective mode areas are determined by input of waveguide cross sections (measured in a scanning electron microscope, SEM) into a finite-element-method (FEM) solver. The wavelength dependence of the second-order dispersion is also calculated using the FEM solver. The Sellmeier equation was used to include the wavelength dependence of the silica refractive index^37^. While bulk silica features anomalous dispersion only for wavelengths beyond 1,270 nm, the geometrical dispersion introduced by the strong optical confinement of the ridge waveguide enables anomalous dispersion at much shorter wavelengths. In particular, the zero crossing of the dispersion (*λ*_ZDW_) can be engineered to occur over a wide range of wavelengths from 557 to 731 nm as is apparent in Fig. 1c,d. Importantly, these lie well below the pumping wavelengths of 830 and 1,064 nm that we employ.

The phase matching condition for the dispersive wave generation satisfies the following equation^38^:


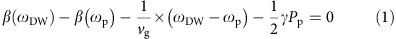


where *β*(*ω*_DW_) and *β*(*ω*_p_) are the propagation constants at the dispersive wave frequency (*ω*_DW_) and the pump frequency (*ω*_p_), respectively, and *v*_g_ is the group velocity at the pump frequency. *P*_p_ is the peak power of the pulse when the dispersive wave is generated. *γ* is the nonlinearity of the waveguide at the pump frequency and is given by *γ*=*ω*_p_*n*_2_/(*cA*_eff_) where *n*_2_ is the Kerr coefficient and *A*_eff_ is the effective mode area. Defining Δ*β*(*ω*)=*β*(*ω*)−*β*(*ω*_p_)−(*ω*−*ω*_p_)/*v*_g_, the dispersive wave phase matching condition is given by Δ*β*(*ω*_DW_)=*γP*_p_/2 and is plotted in Fig. 1d for an 830 nm pump. At this pump wavelength, the phase matching wavelength can be engineered to vary from 310 to 576 nm. Discussion on the range of phase matching wavelengths can be found in Supplementary Fig. 2.

### Imaging dispersive waves in the array

Photographs of pumped waveguides on a single chip are combined in Fig. 1e. The laser pulse in these images is provided by a mode-locked titanium-sapphire laser (830 nm emission, 60 fs pulse width measured on an autocorrelator, and 81 MHz repetition frequency) and is coupled into the silica ridge waveguides using a × 60 objective lens. The coupling efficiency is measured to be 25–35%. As the laser pulse propagates along a given waveguide (left-to-right in image), the infrared pulse is initially invisible. The pulse undergoes temporal compression and spectral broadening because of the self phase modulation and anomalous second-order dispersion. As broadening occurs, portions of the pulse's spectrum becomes visible. Solition fission and dispersive wave generation occur at the bright orange spot. Waveguide areas are larger for upper waveguides in the photograph and decrease towards the lower portion of the plot. Decreasing mode areas shift the phase matching condition to shorter wavelengths so that the dispersive wave generation transitions from the visible to ultraviolet. For wavelengths below 400 nm the dispersive wave is not visible in the photograph.

**Figure 3:**
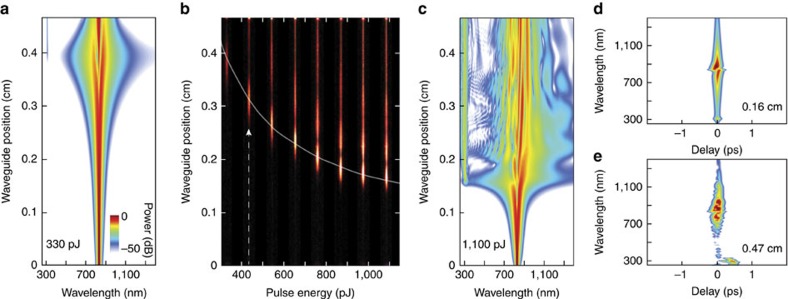
Numerical simulation of pulse propagation in the waveguide and comparision with measurement. (**a**) Calculated continuum spectra as a function of waveguide position at a coupled pulse energy of 330 pJ. The pulse is launched in the TM mode of the waveguide with mode area 0.76 μm^2^. The colour bar (inset) applies to (**a**,**c**–**e**). (**b**) Top-view photographs of scattered light from the surface of the waveguide. The photographs were taken at pulse energies ranging from 330 to 1,100 pJ (left to right side). As indicated by the dashed line, the pulse travels in the waveguide from the bottom to the top of the image. Dispersive wave generation occurs in the ultraviolet and is therefore not visible in the image. The grey line superimposed on the photographs is the length at which dispersive wave generation occurs as predicted by the simulation. The spectral breathing of the input pulse results in a periodically visible orange-red emission that correlates with the calculation. (**c**) Calculated continuum spectra as a function of waveguide position at a pulse energy of 1,100 pJ. (**d**,**e**) Calculated spectrograms of the optical pulse propagating in the waveguide corresponding to (**c**). The spectrogram in **d** is at waveguide position 0.16 cm where dispersive wave is generated. The spectrogram in **e** is at the waveguide output.

More details about the pulse propagation in the waveguide are provided in Fig. 3. Here, calculated spectra are plotted versus propagation length for pulse energies of 330 pJ (Fig. 3a) and 1,100 pJ (Fig. 3c). The pulse initially propagates as a higher-order soliton that undergoes temporal compression and spectral broadening until experiencing soliton fission and dispersive wave generation at a fission length, *L*_*f*_, that depends upon the pulse energy (330 pJ: *L*_*f*_=0.39 cm; 1,100 pJ: *L*_*f*_=0.16 cm). As the input pulse energy increases, the calculated fission length decreases. This behaviour is observable in Fig. 3b where a composite of photographs of the waveguide is shown at coupled pulse energies from 330 to 1,100 pJ. Soliton fission is observable as the reddish-orange emission. The observed soliton fission length agrees well with a numerical simulation shown as the grey line.

The dispersive wave initially overlaps with the soliton pulse but walks off from the soliton with continued propagation because of group velocity mismatch (see Fig. 3d,e). Also, the dispersive wave is temporally stretched as it propagates in the waveguide because of the normal dispersion it experiences. Walk-off is a typical feature of fibre broadeners and requires a delay line to spatially overlap the octave wave (2*f*) and dispersive wave. However, as shown in the next section, the waveguide length can be adjusted to optimize overlap between 2f and dispersive-wave pulses. Moreover, the array, itself, provides the user with the ability to optimize the dispersive wave efficiency around a specific laser source.

### Walk-off-free dispersive-wave-enhanced octave generation

**Figure 4:**
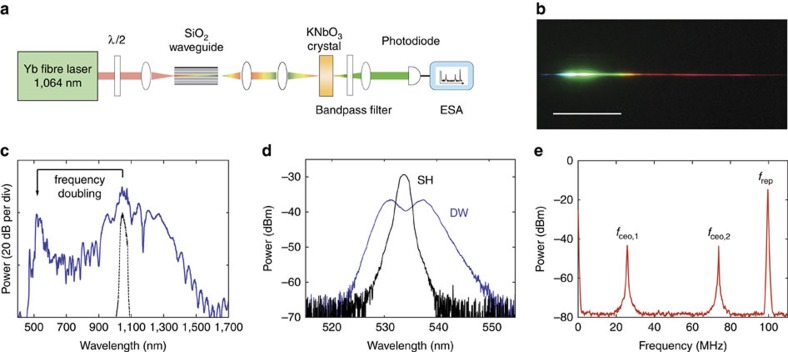
Application of dispersive wave engineering to self-referencing a Yb fibre laser frequency comb. (**a**) Experimental setup for measuring *f*_ceo_ of the Yb fibre laser. The ridge waveguide has a mode area 3.13 μm^2^ and length 1.50 cm. (**b**) Photograph of the emitted light dispersed through a prism and reflected on a white screen. Scale bar, 5 cm. (**c**) Measured optical spectrum of the collimated beam at the output facet of the waveguide. The coupled pulse energy is 2,300 pJ and the input spectrum is shown in black. (**d**) Spectra of the dispersive wave (DW, blue) and second harmonic light (SH, black) filtered by a bandpass filter. (**e**) Radio-frequency spectrum measured with an electrical spectrum analyser (ESA) shows the pulse repetition rate *f*_rep_ and the carrier-envelope-offset beat frequency *f*_ceo,1_ and *f*_ceo,2_. The resolution bandwidth (RBW) is 300 kHz.

Beyond the generation of broadly tunable visible light, precise dispersive-wave engineering in a monolithic waveguide can be applied both to enhance the signal-to-noise ratio as well as simplify the setup for detection of the carrier-envelope offset frequency of a laser frequency comb^3^. As a demonstration, *f*-2*f* offset frequency generation of a Yb fibre laser comb is performed using a silica ridge waveguide. The waveguide having a mode area of 3.13 μm^2^ and length of 1.50 cm is dispersion engineered so as to enhance dispersive wave formation at twice the frequency of the Yb laser comb. The experimental setup is shown in Fig. 4a. The Yb laser emits 90 fs pulses with centre wavelength of 1,064 nm (100 MHz repetition rate). Before waveguide coupling, a halfwave plate rotates the Yb laser polarization so that 80% of the total coupled pulse energy (2,300 pJ) is transmitted in the TE mode and 20% is transmitted in the TM mode of the waveguide.

The TE wave forms a dispersive wave near 532 nm. A photograph of the spectrum of the continuum is shown in Fig. 4b and a spectral scan is provided in 4c. Both the TE and TM waves are subsequently coupled to a potassium niobate (KNbO_3_) crystal where the TM component is phase matched for second harmonic generation. The phase-matching condition generates the second-harmonic beam that is rotated by 90° relative to the TM fundamental so that the second-harmonic polarization is aligned with the TE polarized dispersive wave. The measured doubled spectrum and the dispersive-wave spectrum are shown in Fig. 4d. After spectral filtering around 532 nm the two waves are mixed on a photodiode for offset frequency (*f*_ceo_) beatnote generation. In this final step, the enhanced dispersive wave comb lines increase the beatnote strength. The electrical spectrum of the beatnotes at *f*_ceo,1_=25.8 MHz and *f*_ceo,2_=*f*_rep_−*f*_ceo,1_=73.8 MHz are shown in Fig. 4e. Both signals have a signal-to-noise ratio >34 dB at an electrical resolution bandwidth of 300 kHz. This establishes the coherent nature of the dispersive wave and is more than sufficient for subsequent self-referenced servo control of the comb.

Beyond the enhanced signal-to-noise provided by the ability to select an optimal waveguide in the array for matching to the 2*f* frequency, there is a key simplification enabled by colinear generation of dispersive and second harmonic waves. The overall group velocity dispersion between the TE dispersive wave and the TM wave at 1,064 nm in both the waveguide array and the KNbO_3_ crystal is such that both the dispersive wave and the second-harmonic pulses emerge from the doubling crystal with a high degree of spatial overlap (that is, walk-off-free). As a result, a major simplification is possible by eliminating the traditional step of path-length balancing using a Michelson interferometer. Instead, the two pulses (the dispersive wave and the second-harmonic pulse) can be directly coupled to the photodetector^39^.

### Spectral measurements

To further probe the behaviour of the dispersive wave generation process, spectral measurements were performed at varying waveguide cross sectional areas. For these measurements, the titanium sapphire laser was used (see earlier discussion) and the experimental setup included an attenuator to vary the input pulse energy, as well as a half-wave plate to control polarization. The light output from the waveguides is endfire-coupled to a multimode fibre for the measurement on a spectrometer. To cover the entire spectral range of interest two spectrometers were used: a Yokogawa (AQ6370D, 600–1,700 nm) and an Ocean Optics (HR4000, 200–900 nm).

**Figure 5:**
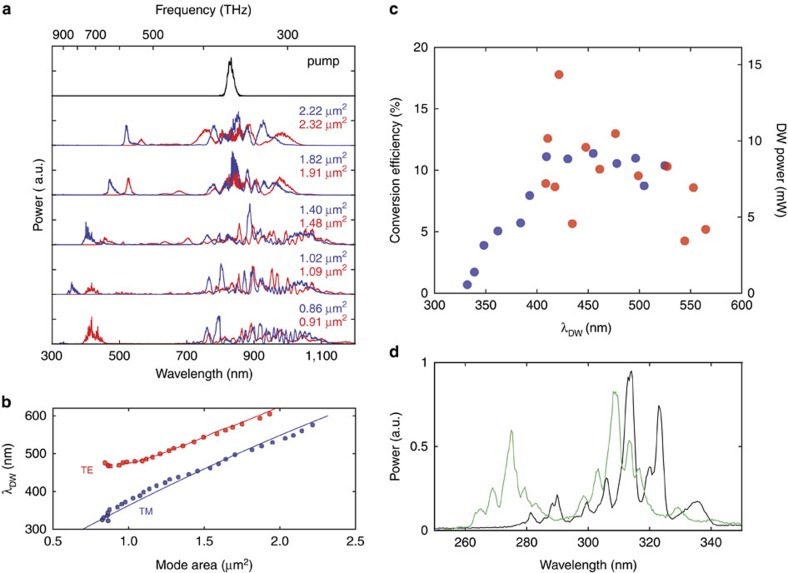
Supercontinuum spectra and the conversion efficiency of near-infrared pump into ultraviolet–visible wavelengths. (**a**) Supercontinuum spectra for a series of ridge waveguides on a single waveguide array chip shown in Fig. 1e when the titanium sapphire laser pulse is coupled to the TM mode (blue line) and TE mode (red line) of the waveguides. The coupled pulse energy is 1,000 pJ. The mode area of the waveguide is indicated in the panel. All the continuum spectra are normalized such that the areas under the spectra are the same. (**b**) Measured tuning of the dispersive wave peak wavelength (*λ*_DW_) by lithographic control of the waveguide mode effective area. The data are taken for a pulse energy at the dispersive wave generation threshold. The solid line is the phase matching condition obtained from FEM simulations. (**c**) Scatter plot of measured conversion efficiency (left axis) and dispersive-wave average power (right axis) versus the dispersive wave peak wavelength for the waveguide array in Fig. 1e. Blue and red markers correspond to the TM mode and the TE mode. The coupled pulse energy is 1,000 pJ. (**d**) Measured dispersive wave spectra in deep ultraviolet when the Yb laser pulse is launched into the TM mode of 0.75 cm-long ridge waveguides having mode areas of 1.09 μm^2^(green) and 1.12 μm^2^ (black). The coupled pulse energy is 2,000 pJ.

Measured supercontinuum spectra are shown in Fig. 5a for TM and TE polarized pulses launched into waveguides of varying mode area in the waveguide array shown in Fig. 1e. Conversion of pump light in the near infrared to visible and ultraviolet wavelength dispersive waves is apparent in each spectrum. A summary of measured dispersive wave emission wavelengths *λ*_DW_ versus mode area is provided in Fig. 5b. Controlled tuning of the dispersive wave from 310 to 576 nm with an average 8 nm interval is demonstrated in the TM mode and from 475 to 613 nm in the TE mode. Also included in Fig. 5b is the predicted dispersive wave emission wavelength using the phase matching condition (equation (1)). The measurement result agrees well with the calculation.

To investigate the conversion efficiency of the input pulse from the near-infrared into ultraviolet–visible wavelengths (300–650 nm), a ultraviolet fused silica aspheric lens (transmission 200–2,000 nm) was used to collect the light exiting the waveguides. The optical power in the ultraviolet–visible wavelengths was then filtered out with a bandpass filter and measured by a thermopile power metre (nearly uniform spectral response). The power was calibrated using the wavelength-dependent transmission curves of the bandpass filter and the measured supercontinuum spectra. The scatter plot of measured conversion efficiency (and average dispersive wave power) plotted versus dispersive wave peak wavelength for both polarizations is shown in Fig. 5c. For this measurement, conversion efficiency is defined as the dispersive wave power at the waveguide output divided by the total collected spectral power at the waveguide output. The spectral extent of the dispersive wave was defined by selecting wavelengths where the power spectral density of the dispersive wave had fallen to 5% of the maximum value.

Using the Yb laser emission at 1,064 nm, it was also possible to investigate even deeper ultraviolet dispersive wave generation below 300 nm. This observation is consistent with the phase-matching condition for dispersive wave generation (Supplementary Fig. 2). Figure 5d shows supercontinuum spectra measured in the TM mode of two waveguides having cross-sectional areas of 1.09 and 1.12 μm^2^ using a coupled pulse energy of approximately 2,000 pJ. The spectra feature multiple peaks because of soliton breathing and subsequent dispersive wave emission. The shortest wavelength edge of the spectrum is nearly 265 nm for the case of the waveguide with mode area 1.09 μm^2^.

## Discussion

A silicon chip based waveguide array has been applied to generate ultraviolet to visible light by conversion of an input pulse into a dispersive wave. The dispersive wave emission wavelength is precisely tuned from ultraviolet to visible by lithographic control of the waveguide dimensions. Generation of ultraviolet emission as short as 265 nm has been demonstrated. The measured and predicted dispersive wave emission wavelengths are in excellent agreement. Arrays-on-a-chip containing hundreds of waveguides are easily fabricated and provide ready access to a range of emission wavelengths using a single pump laser. This chip-based tuning control allows for optimization of emission for spectroscopy and metrology using a single device. As a demonstration, offset frequency generation in a Yb mode-locked laser frequency comb was demonstrated by designing a waveguide to generate a dispersive wave that is optimally matched to the second harmonic of the original 1 μm comb. Significantly, this demonstration also confirms the high coherence of the dispersive wave generated by these waveguides. Moreover, the ability to tailor the chip length to provide walk-off-free self-referencing was demonstrated. Monolithic waveguide arrays for wide-band coherent optical generation (up to two octaves) provide a new capability for integration with other optical elements on a chip and can also find application in other areas including bioimaging.

## Methods

### Dispersion characterization

To verify that the waveguide exhibits anomalous dispersion near the value predicted by the FEM simulations in Fig. 1d, the group velocity dispersion is characterized using a Mach-Zehnder interferometer having one arm with an adjustable delay^40^. In the dispersion measurement, a probe pulse is attenuated to a low enough pulse energy (<30 pJ) so as to prevent significant nonlinear phase shifts in the waveguide. The pulse is split using a beamsplitter and propagates into two arms of a Mach-Zehnder interferometer. One pulse is coupled to the waveguide and the other one propagates through the adjustable delay line in the air. The light emitted from the waveguide is collimated and the pulses from the two arms are combined using a second beamsplitter. The combined beams are then focused into a single mode fibre and sent to a spectrometer. The time delay between the two pulses is calculated using the Fourier-transform of the measured spectrum which shows spectral fringes^41^. By tuning the laser wavelength from 770 to 810 nm, the dispersion of the waveguide is then measured from the observed wavelength-dependence of the group delay in the waveguide arm. The measured dispersion is plotted in Fig. 1d and agrees well with the FEM simulation. The error bars in Fig. 1d are derived from the standard deviation of group delay from 10 spectral measurement at each wavelength.

### Numerical simulations

Numerical simulations of pulse propagation in the waveguides were performed using a generalized nonlinear Schrödinger equation. To model the frequency dependence of the nonlinear response, the following equation is used^42^.


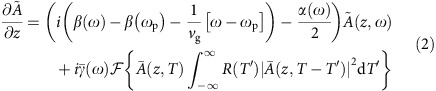


Here, 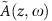 is the complex spectral envelope of the pulse at waveguide position *z*, 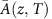 is defined as 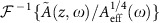, *α*(*ω*) is the linear loss and *R*(T) is the Raman response function. 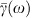 is the parameter related to the nonlinear response defined as:


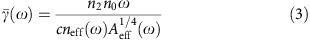


The frequency-dependent propagation constant (*β*(*ω*)), effective mode index (*n*_eff_(*ω*)) and mode area (*A*_eff_(*ω*)) of the waveguides were calculated using FEM simulations and imported into the pulse propagation simulations.

### Data availability

The data that support the plots within this paper and other findings of this study are available from the corresponding author on reasonable request.

## Additional information

**How to cite this article:** Oh, D. Y. *et al*. Coherent ultra-violet to near-infrared generation in silica ridge waveguides. *Nat. Commun.* **8,** 13922 doi: 10.1038/ncomms13922 (2017).

**Publisher's note**: Springer Nature remains neutral with regard to jurisdictional claims in published maps and institutional affiliations.

## Supplementary Material

Supplementary Information Supplementary Notes and Supplementary Figures
