# Supplementary material for: Coherent ultra-violet to near-infrared generation in silica ridge waveguides
Source: Nat Commun. 2017 Jan 9;8:13922. doi: 10.1038/ncomms13922 (PMC5227738; doi:10.1038/ncomms13922)
Supplement: Supplementary Information — Supplementary Notes and Supplementary Figures [file ncomms13922-s1.pdf]

Supplementary Note 1

Waveguide arrays are fabricated on (100) prime-grade float-zone silicon wafers. The initial oxide layer is thermally grown at 1000°C with 2 μm thickness. The photoresist is patterned on the oxide layer (Fig. 2a), and acts as etch mask during hydrofluoric acid (HF) immersion. HF wet-etching creates the wedge surfaces at the edge of the photoresist pattern, and the further wet-etching results in the triangular-cross-section ridge stripe of silica as the two angled wedge surfaces meet each other (Fig. 2b). The wet-etching duration is around 45 min. Then, an additional thermal oxidation creates an under-layer of silica (Fig. 2c). The waveguide chips used for data in the manuscript had the under-layer thickness of either 310 nm or 450 nm. Striped openings (Fig. 2d) are etched after a second lithography step (Fig. 2e). As a final step, the silicon under the oxide structure is isotropically etched (Fig. 2f). Both numerical calculation and measurement confirmed that an undercut of 10 μm is sufficient to eliminate the silicon structure interaction as a result of modal confinement. The average spacing between two waveguides is about 35 μm, and 725 waveguides per inch can be fabricated in an array.

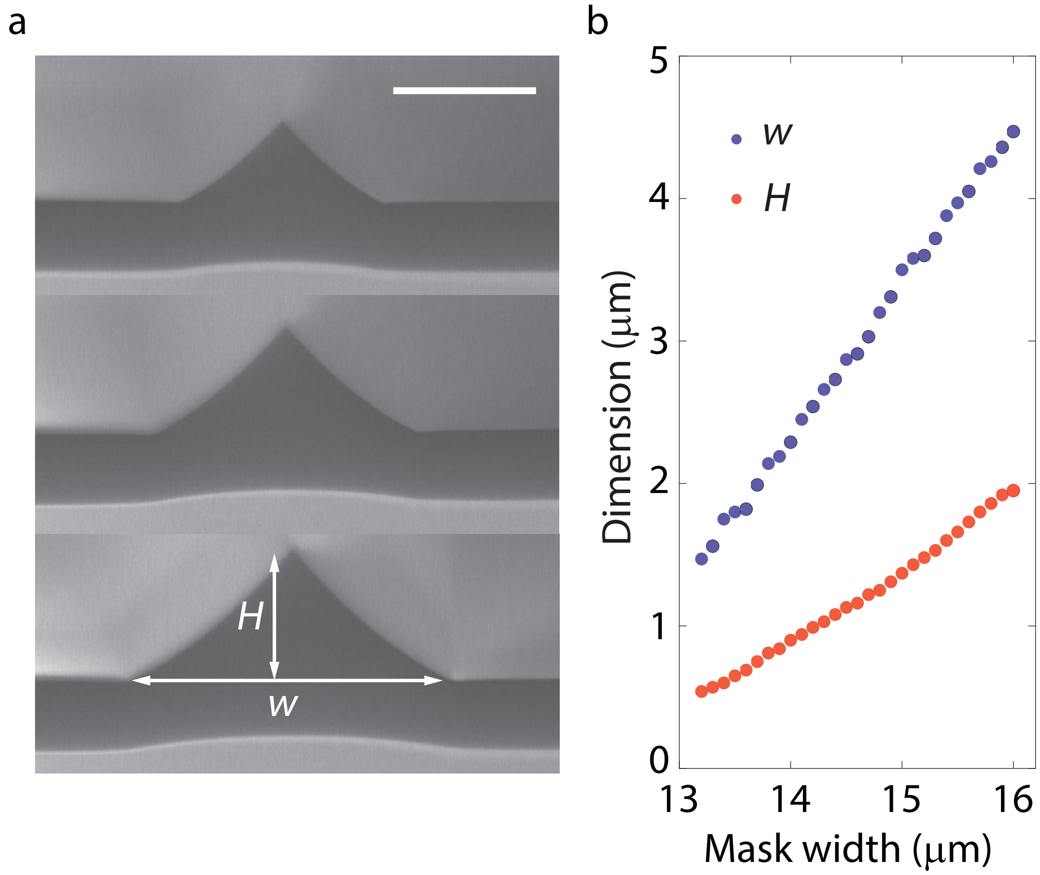

Supplementary Figure 1: Dependence of ridge dimension on mask width (**a**) SEM images of a series of ridges in a fabricated silica waveguide array. The thickness of the base silica layer is 0.45 μm. Scale bar is 1 μm. The definition of ridge height (H) and ridge width (w) is shown in the panel. (**b**) Measured ridge dimensions based on SEM images as a function of mask width. Here, the mask width is the width of the red rectangular strip patterned on photoresist in Fig. 2a of the manuscript. The ridge height (H) and width (w) increase by about 0.5 μm and 1 μm, respectively, as the mask width increases by 1 μm. The ability to lithographically define the ridge dimension enables precise control of waveguide dispersion, and, in turn, precise tuning of the dispersive-wave wavelength.

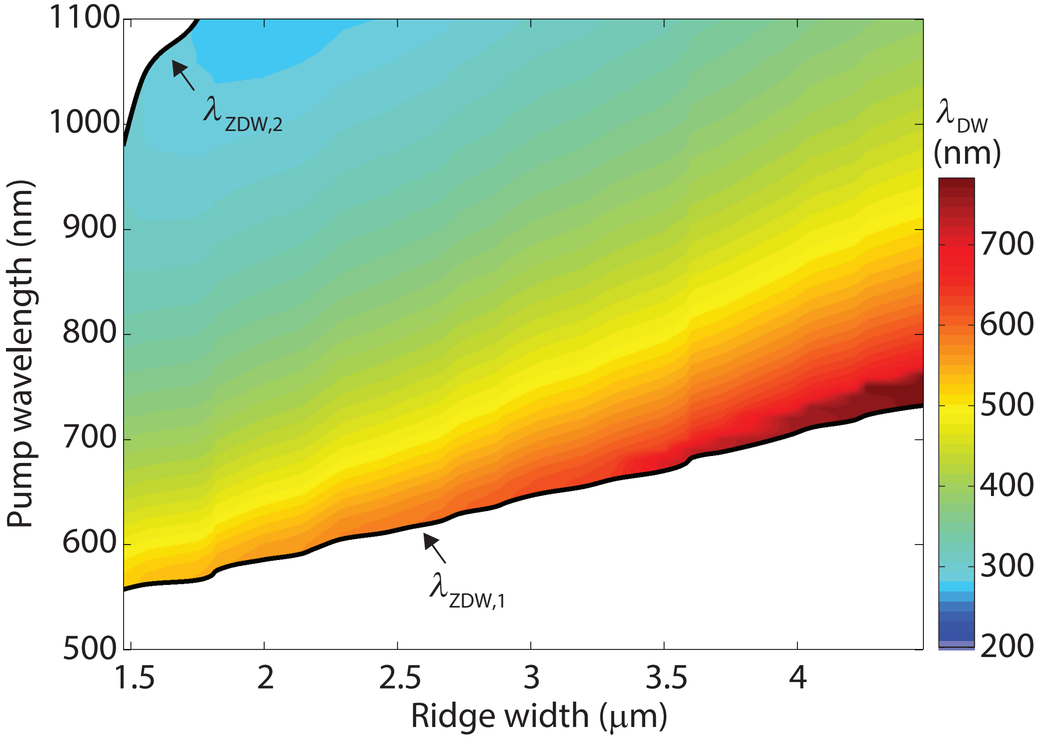

Supplementary Figure 2: Phase-matching dispersive wave wavelength in a waveguide array. Calculated TM dispersive wave wavelength given as a colour map (λ_{DW}) as a function of pump wavelength and ridge width for the waveguide array chip used in Fig. 1e. The black lines are the zero crossing of the group velocity dispersion ($\lambda_{\mathrm{ZDW},1}$ and $\lambda_{\mathrm{ZDW},2}$). The white regions are pump wavelengths where dispersive wave generation is not possible. For waveguides with $w < 2.19$ μm, there is a second zero crossing ($\lambda_{\mathrm{ZDW},2}$), allowing for formation of a second dispersive wave. However, a second dispersive wave was not observed in our experiment due to the loss of modal confinement at long wavelengths for the waveguides with small mode area. An upper bound on λ_{DW} for a given ridge width is provided by $\lambda_{\mathrm{ZDW},1}$. λ_{DW} will approach this wavelength as the pump wavelength approaches $\lambda_{\mathrm{ZDW},1}$. On the other hand, a lower bound on λ_{DW} is determined by the longest pump wavelength possible in the anomalous dispersion regime (coloured region). λ_{DW} shifts toward shorter wavelength as the pump wavelength increases. This is understood from the phase-matching condition. At longer pump wavelengths, the group velocity is smaller, and hence the propagation constant of the dispersive wave must be larger (shorter wavelength) for the phase-matching to occur. The upper bound on the pump wavelength in the anomalous dispersion regime is given by $\lambda_{\mathrm{ZDW},2}$ if it exists. The calculation is consistent with our demonstration of dispersive wave generation at wavelength $\lambda_{\mathrm{DW}} < 300$ nm using a 1064 nm pump wavelength as compared to the shortest λ_{DW} of 310 nm generated using an 830 nm pump wavelength. Lastly, smaller mode area results in smaller λ_{DW} and can be achieved with smaller ridge widths. However, there is a threshold value of ridge width below which the mode area increases because of weak modal confinement. For the array waveguide chip with lower silica layer thickness 0.45 μm, the threshold ridge width is 1.47 μm using the 830 nm pump.
